# Effects of L-carnitine and L-acetyl-carnitine on testicular sperm motility and chromatin quality

**Published:** 2012-03

**Authors:** Elham Aliabadi, Malek Soleimani Mehranjani, Zahra Borzoei, Tahereh Talaei-Khozani, Hossein Mirkhani, Hamed Tabesh

**Affiliations:** 1 *Department of Anatomy, Shiraz University of Medical Sciences (SUMS), Shiraz, Iran. *; 2 *Department of Biology, Faculty of Sciences, University of Arak, Arak, Iran. *; 3 *Department of Pharmacology, Shiraz University of Medical Sciences, Shiraz, Iran. *; 4 *Department of Biostatistics, Shiraz University of Medical Sciences, Shiraz, Iran. *

**Keywords:** *Sperm*, *L-Carnitine*, *L-Acetyl-carnitine*

## Abstract

**Background:** Sperm cells extracted from testes (TESE) have poor chromatin quality and motility. Various substances are used in the laboratory to increase sperm motility and improve the ART outcomes; however, there are few research which considered improving both sperm motility and chromatin quality.

**Objective:** The aim of this investigation was to evaluate the improvement of the testicular sperm motility and chromatin quality exposed to L-carnitine (LC) and L-acetyl-carnitine (LAC), which are normally concentrated in testis and epididymis, compared with Pentoxifylline (PF), which used for sperm motility enhancement in IVF procedures.

**Materials and Methods:** TESE samples from 30 male mice divided into four parts. The sperm samples were added to Ham' F10 (control) or the media contained 1.76mM of LC, LAC or PF), then, the samples were kept in the room temperature for 30, 90 and 180 min. At each time step, sperm motility and chromatin quality were assessed. Chromatin quality was evaluated by chromomycin A3 and aniline blue. Statistical analysis was performed using one way analysis of variance (ANOVA). A p-value less than 0.05 were accepted as a statistically significant difference.

**Results: **The results showed LC, LAC and PF significantly increased the sperm motility. However, sperm chromatin quality only improved significantly by administration of LC and LAC.

**Conclusion:** Administration of LC and LAC to the testicular sperm samples can lead to improve both sperm motility and chromatin quality. It may be because they can mimic in vivo sperm condition during late spermatogenesis.

## Introduction

Thirty to fifty percent of all cases of infertility occurred due to the male factors. In 30% of infertile couples, the most common reason is the deficiency in sperm motility. Sperm quality is impaired by several abnormal conditions during spermatogenesis (1). Non-obstructive azoospermia patients have no chance to be fertile in a normal intercourse. They have to be involved in an ART procedure (2).

In mammals, including human beings, most of the spermatozoa leave the testis are infertile. The spermatozoa become fertile after several successive biochemical changes while they cross through male and female genital tracts (3). Chromatin quality and motility changes as they transit along the epididymidis (4). It should be considered that the motility and chromatin quality of fresh biopsied testicular spermatozoa is poor and they often demonstrate a non-progressive movement. The morphology and motility of fresh testicular spermatozoa can be significantly improved after in-vitro culture. However, it takes several days for them to be matured (5). 

Improving the fertility rate by choosing a good quality sperm is the goal of many ART investigations. Maturation of the sperm extracted from testis is an important point for successful ART program in TESE patients. Epididymis secrets many chemicals such as L-carnitine (LC) and L-acetyl-carnitine (LAC) that involved in sperm maturation (3). Biological roles of the epididymis in sperm maturation are considered in some research (4, 6). L-carnitine, with extensive physiological roles is essential nutrient for the body health. It is concentrated in the epididymis and sperm (7). Despite blood-testis barrier, carnitine is also highly concentrated in testis (8). 

“It plays an important role not only in initiating sperm motility, promoting sperm maturation and enhancing sperm fertilizing, but also in regulating Sertoli cell functions and protecting sperms against oxidative damage, reducing apoptosis of spermatogenic cells and inhibiting sperms aggregation” (9). Improvement in sperm chromatin quality was demonstrated by oral intake of some anti-oxidant agents such as LC (10). Protamine packaging was also influence by oxidative stress and administration of antioxidant improved protamination that was revealed by chromomycin A3 (11). 

The oxidative stress causes DNA damage. DNA damage was correlated with DNA protamination during late spermatogenesis (12). In the light of these considerations, we hypothesized that these chemicals can be added to the culture medium of the testicular sperm to improve sperm motility and chromatin quality in late spermiogenesis. With regards to the roles of carnitine in mitochondrial lipid peroxidation and its anti-oxidative effects, this study was designed to investigate the effects of L-carnitine and L-acetyl-carnitine on testicular sperm motility and chromatin quality in vitro.

## Materials and methods

**Animals**

The study design was experimental intervention. Thirty mature male balb/C mice weighting 30-35 g were acclimated to the *laboratory* condition (12 h light, 12 h darkness and temperature 24 C). 

**Sperm preparation**

The testes removed from each mature male mouse under deep anesthesia by diethyl ether. The testes were rinsed with Ham’s F10. After the removal of the tunics, seminiferous tubules were separated by two needles gently. 

To separate red blood cells, 3 mL of culture media was added and centrifuged at 500 rpm for 10 min. Red blood cells were appeared as a thin layer on the top of the tube removed by a pipette. The remaining tissue was removed to a Petri dish containing 3 mL of culture media and splice into several pieces. The seminiferous tubules were vortexed for 60 seconds to extract the spermatozoa from the tubules (13). The sample was incubated at room temperature for 1 h (14) then centrifuged at 500 rpm for 10 min. Leydig cells, sertoli cells and connective tissue were precipitated. The supernatant was centrifuged again at 2000 rpm for 10 min. The pellet contained sperm (13), was suspended in 1 mL of culture medium. 

**Study groups**

Sperm samples divided into several parts as follow: 0.2 mL of sperm sample was added to 0.2 mL of Ham’s F10 (control) (Sigma, USA). In treatment groups, 0.2 mL of sperm sample was added to 0.2 mL of Ham’s F10 containing 3.6 mM of L-carnitine (Sigma, USA) (LC group) or L-acetyl-carnitine (Sigma, USA) (LAC group). In positive control group, 0.2 mL of sperm sample was added to 0.2 mL of Ham’s F10 containing 3.6 mM Pentoxifylline (Sigma, USA) (PF group). Therefore, the final concentration of 1.76 mM was obtained (15). Sperm motility and chromatin quality were evaluated at 30, 90 or 180 min after incubation at the room temperature; then, the results were compared with control specimens.

**Sperm motility assay**

Sperm smears from all specimens were prepared at 30, 90 and 180 min after incubation. For evaluating motility, "sperm cells were classified as immotile (IM, no movement), non-progressive motile (NP, all other patterns of motility with an absence of forward progression, e.g. swimming in small circles, the flagella force hardly displacing the head, or when only a flagella beat can be observed) and progressively motile (PR, spermatozoa moves actively, either linearly or in a large circle, regardless of the speed)" (16). To calculate the motile sperm percentage, 100 of sperm cells were counted in each slide (17). 

**Chromatin assay**

**Figure 1:**
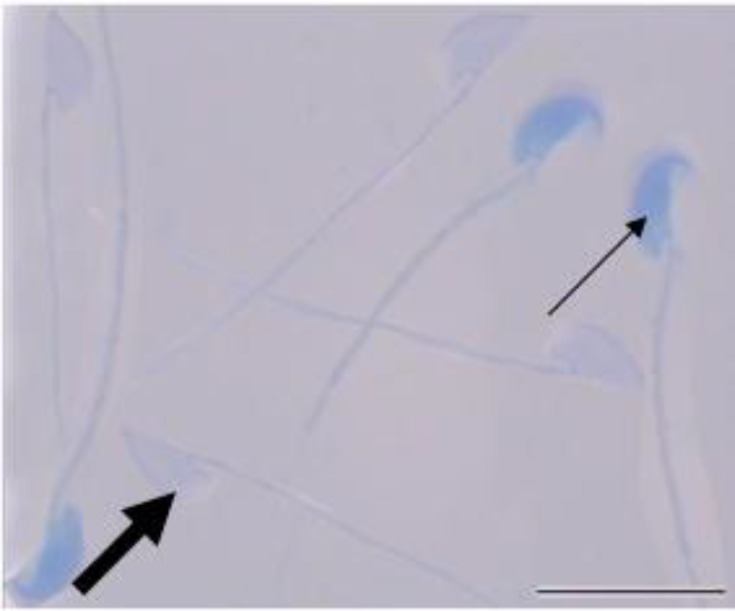
Aniline blue stains histone content of sperm nuclei. More intense staining indicates immature sperm (thin arrow). The mature (normal) spermatozoa stain weak with aniline blue (thick arrow). Scale bar is 10μm

**Figure 2:**
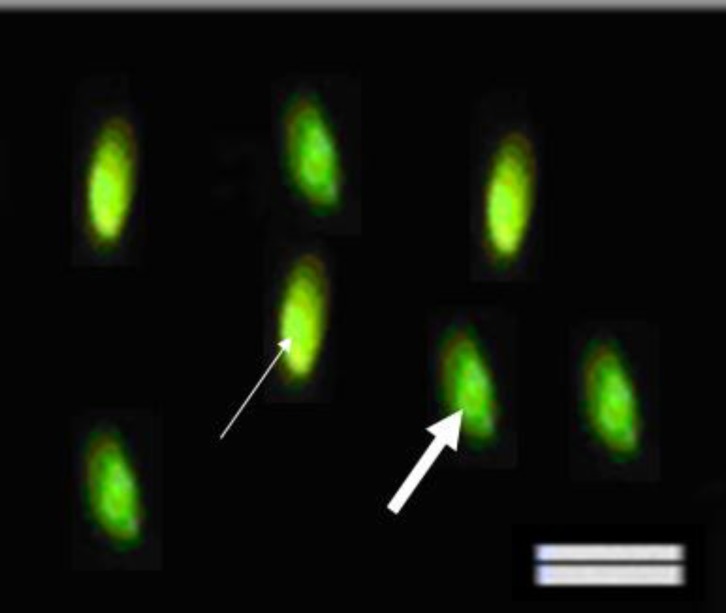
Chromomycin A3 reacts with protanine content in sperm nulclei. The abnormal sperm excites yellow fluorescence (thin arrow) and the normal sperm (thick arrow) excites green fluorescence and. Scale bar is 10μm

Sperm smears from all groups were prepared at 30, 90 and 180 min after incubation. For evaluating sperm chromatin quality, aniline blue and chromomycin A3 staining techniques were performed. These techniques detect the amount of histone (18) and protamine (19), respectively. The percentage of stained spermatozoa was calculated **(**Fig 1** and **2**) **the percentages of aniline blue (AB) and chromomycin A3 (CMA3)-positive sperm cells were counted. More intense staining with AB (dark blue), shows more histone content in the sperm chromatin. Shiny yellow CMA3 fluoresces shows less protamination degree in sperm nuclei. 

**Statistical analysis**

All results were presented as mean±S.E (standard error of mean). Statistical analyses were performed using one way analysis of variance (ANOVA). A p-value less than 0.05 were considered as significant difference. 

## Results

Motility assay showed a significant decrease in percentages of immotile cells and a significant increase in the percentage of the progressive motile cells in LC and LAC-treated samples compared to the control sample at 30, 90 and 180 min after incubation (p<0.05) (Table I). Aniline blue test showed a significant decrease in the percentage of AB positive cells only in LAC-exposed spermatozoa compared to the control sample at 180 min after incubation (p<0.05) (Table I, Figure 1). 

Chromatin assay with CMA3 showed a significant decrease in the percentage of CMA3 positive cells in LC and LAC-treated samples compared to the control sample at all time steps (p<0.05) (Table I, Fig 2). At 30, 90 and 180 min after incubation, there was a significant decrease in the percentage of immotile spermatozoa in PF-treated samples compared to the control samples (p<0.05). 

At the same times, the percentages of the progressive spermatozoa of the same samples were also increased significantly; however, the percentage of non-progressive cells increased compared to the control sample at 90 min after incubation. There was no significant difference between the percentages of motile, non-progressive and immotile spermatozoa in other samples. 

Motility test showed, a significant increase in the percentage of progressive spermatozoa in the PF-treated cells compared to the LC-treated cells after all time steps and to the LAC-treated cells at the 30 min of incubation (p<0.05). At 90 min after incubation, the PF-treated cells showed a significant decrease in the percent of non-progressive spermatozoa compared to the LC-exposed cells (p<0.05) (Table I). 

Chromatin assay with CMA3 showed a significant increase in the percentage of CMA3-positive cells in PF-treated spermatozoa compared to the LC and LAC-treated cells at all time steps (p<0.05) (Table I). Administration of the PF for 180 min to the cultures revealed a significant increase in AB-positive spermatozoa compared to the LAC-treated cells (p<0.05) (Table I).

**Table I:** Effects of the L-carnitine, L-acetyl carnitine and pentoxifylline on the motile spermatozoa percentages after incubation for 30, 90 and 180 min (n=30).

		**IM (%)** **Mean±S.E**	**NP (%)** **Mean±S.E**	**PR (%)** **Mean±S.E**	**AB** ^+^ ** (%)** **Mean±S.E**	**CMA3 (%)** **Mean±S.E**
Control						
	30	36.23±3	37.83±3	25.83±3	44.1±2	61.73±3
	90	32.53±3	38.8±3	28.33±3	44.23±2	59.03±3
	180	36.56±3	34.26±3	30.03±3	40.16±2	54.86±3
L–carnitine group (LC)						
	30	27.73±2٭	36.6±2	35.76±2٭ †	43.97±3	56.97±3٭ †
	90	18.96±2٭	38.76±2†	42.26±3٭ †	38.77±2	51.4±3٭ †
	180	20.7±3٭	36.76±3	42.53±3٭ †	38.1±2	46.43±2٭ †
L–acetyl-carnitine group (LAC)						
	30	25±3٭	34±1	41.16 ±3٭ †	46.47 ±2	57.1 ±3٭ †
	90	16±2٭	32.16±2٭	51.5 ±3٭	42.03 ±2	49.53 ±3٭ †
	180	14.4±3٭	27±2	58.36 ±3٭	34.26 ±2٭ †	45.23 ±3٭ †
Pentoxifylline group (PF)						
	30	20.4±2٭	30.3±2	49 ±3٭	50.5 ±2	62.06 ±3
	90	15.13±2٭	28.4±2٭	56.2 ±3٭	44.83 ±3	57.57 ±3
	180	17.26±2٭	30.96±2	51.86 ±3٭	43.8 ±1	55.83 ±3

Immotile (IM);

Non- progressive (NP);

progressive (PR);

Anilline blue positive cells (AB+): Sperm heads with more intense reaction showed immature sperms with more histone content;

Chromycine A3 positive cells (CMA3+): Sperm heads with yellow florescence reaction showed protamination deficiency.

* Significant difference with control (p< 0.05).

† Significant difference with PF (p<0.05).

## Discussion

A semen contains high qualified spermatozoa is a necessity to have a successful ART protocol. Many research are performing to improve semen quality by adding chemicals to culture media or taking it as a drug. Chemicals such as LC, LAC (20) and PF (14) can improve sperm parameters that are critical for fertilization and embryo development. As the research showed, PF caused an increase in testicular sperm motility (20). The data presented by this research also confirm previous studies. 

Tasdemir *et al* reported PF increases sperm motility via controlling the activity of phosphor diesters enzyme and increasing intracellular level of cAMP and therafter glycocysis and energy production (21). Our data indicated administrations of LC and LAC to the culture media also lead to increase in testicular sperm motility. 

Lenzi *et al* demonstrated that daily diet containing LC and LAC lead to a significant increase in sperm motility in the men who suffer from oligo as the noteratozoospermia (20). Tanphaichiter has also reported higher percentage of motile ejaculated sperm cells in patients with astenospermia exposed to LAC (22). Improvement of sperm motility was also shown after in vitro exposure of testicular sperm to LC (23). Our data confirmed these results.

Free radical oxygen species (ROS) in culture media can disturb sperm motility via interruption with ATP production or flagellar axonome phosphorylation (24). Anti-oxidant property of LC and LAC may also have influence in sperm motility. L-carnitine and LAC, as anti-oxidant (25), may protect sperm plasma membrane with high level of unsaturated fatty acid content (26). 

Free radicals can also decrease mitochondrial energy availability and impaired sperm motility (27). L-carnitine increases sperm motility by changing in fatty acid metabolism (28). Mitochondria in the middle piece of the sperm involve in fatty acid metabolism. L-carnitine and LAC act as buffering system to adjust acetyl-CoA concentration (29). The presence of acetyl-CoA is essential for tricarboxylic acid cycle and energy production. It has been postulated that both of the chemicals influence respiratory reactions and energy production by regulating acetyl-CoA concentration (30). 

Pentoxifylline has been demonstrated to increase testicular sperm motility when it added to culture media (14). As our data indicate, the impact of PF in testicular sperm motility is time dependent. However, LC and LAC lead to an increase in sperm motility compared to the control sample without time limitation. It may be because PF is toxic and exposure time more than 90 min is not recommended (14); While, LC and LAC are amino acids that present in normal microenvironment of the testis (8). Our data showed it took longer time for LC and LAC than PF to increase motility. It may be due to the various energy sources (29, 30) that spermatozoa use after exposure to the chemicals. 

 Sperm genetic material is also important criterion for success of ART programs (31). The testicular spermatozoa are immature and should continue their maturation process through the late spermiogenesis stages including completion of protamination. Transcription of protamine gene was occurred during spermiogenesis (32). Histone can convert into protamine by histone hyperacetylation (33). Literature showed chromatin condensation, assessed by aniline blue staining, was significantly higher in the human ejaculated spermatozoa than in testicular sperm (34). In vitro administration of LC to the human testicular sperms can regulate protamine expression in a dose dependent manner (23). Our data also confirm these findings.

Free radicals are formed during sperm preparation and washing. High level of ROS in the sperm medium causes breakdown of nuclear and mitochondrial DNA (26). A correlation between presence of oxidative stress in infertile men and sperm DNA damage was reported previously (35). Antioxidant treatment improves sperm DNA damage (36). DNA damage may be resulted from abnormal chromatin packing (37). Protamine packaging was also influence by oxidative stress (11). The results of this study showed LC and LAC, as anti-oxidant agents, could improve the percentages of the testicular spermatozoa with less histone and more protamine content. 

Besides, the research showed histone hyperacetylation plays an important role in substitution of histones by protamines. L-acetyle carnitine is considered as donor of acetyl group and can transfer its acetyl group to histone by acetyl transferase enzyme and by this way it can improve protamination (38). 

## Conclusion

The results of the present study indicate that:

1) L-carnitine and LAC led a significant increase in the percentage of the motile testicular sperm. The administration of the chemicals also showed a significant increase in the percentage of spermatozoa with normal histone and protamine content. 

2) Pentoxifylline caused a significant increase in motile testicular sperms; however, PF could not influence sperm chromatin quality.

3) Therefore, it seems that LC and LAC can be a suitable substitution for PF to improve mouse sperm motility and chromatin quality in vitro. 

## Footnote

***This article was taken from the master’s thesis for the M.Sc. degree***
